# Strategies for designing low thermal quenching upconverting temperature sensors

**DOI:** 10.1039/d3ra01679j

**Published:** 2023-05-25

**Authors:** Manisha Prasad, Vishab Kesarwani, Vineet Kumar Rai

**Affiliations:** a Laser and Spectroscopy Laboratory, Department of Physics, Indian Institute of Technology (Indian School of Mines) Dhanbad-826004 Jharkhand India vineetkrrai@yahoo.co.in vineetkrrai@iitism.ac.in +91-326-223-5404 extn 5282

## Abstract

The Er^3+^/Yb^3+^:NaGd(WO_4_)_2_ phosphors and the phosphor-in-glass (PIG) have been synthesized employing a typical approach to investigate their structural, morphological and optical properties. Several PIG samples containing different amounts of NaGd(WO_4_)_2_ phosphor have been manufactured by sintering the phosphor and glass [TeO_2_–WO_3_–ZnO–TiO_2_] frit together at 550 °C, and its impact on the luminescence characteristics has been extensively studied. It has been noticed that the upconversion (UC) emission spectra of PIG under 980 nm excitation display similar characteristic emission peaks to the phosphors. The maximum absolute sensitivity of the phosphor and PIG is 17.3 × 10^−3^ K^−1^ @ 473 K and the maximum value of relative sensitivity is 10.0 × 10^−3^ K^−1^ @ 296 K and 10.7 × 10^−3^ K^−1^ @ 298 K, respectively. However, thermal resolution at room temperature has been improved in the case of PIG as compared to the NaGd(WO_4_)_2_ phosphor. As compared to the Er^3+^/Yb^3+^ codoped phosphor and glass, the less thermal quenching of luminescence has been observed in PIG.

## Abstract 2

Security application of Er^3+^/Yb^3+^ doped NaGd(WO_4_)_2_ phosphor upon 980 nm excitation and absolute and relative sensitivity of Er^3+^/Yb^3+^ doped NaGd(WO_4_)_2_ phosphor and PIG.

## Graphical abstract

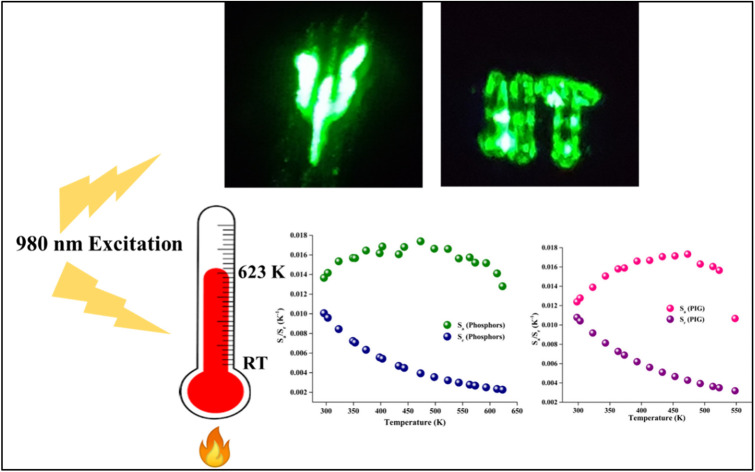

## 1. Introduction

The existing era of luminescence-based applications is based mainly on rare-earth (RE) doped materials owing to their high luminescence efficiency, long lifetime, low power consumption, excellent stability, and flexible control of photometric properties.^1–5^ Several methodologies have been tried by the researchers to develop new types of RE-doped luminescent materials for better results in various applications. The RE-doped luminescent materials have two categories, *i.e.*, downconverting and upconverting luminescent materials. Among these, the upconverting luminescent materials have some advantages over the downconverting ones, such as the absence of background autofluorescence, high penetration depth and cheap excitation source.^6^ The upconverting materials have shown encouraging results in applications including optical temperature sensing, solar cells, photodynamic therapy (PDT), and bioimaging.^7–10^ In search of better results, researchers have tried to synthesize different types of functionalized upconverting nanoparticles (UCNPs) such as core@shell NPs, dye-sensitized UCNPs, and heavily doped UCNPs for various applications.^11^ One such approach that is attracting researchers nowadays is phosphor-in-glass (PIG) based luminescent materials.

PIG, a mixture of a RE-doped phosphor and transparent glass sintered at low temperature (usually <800 °C), has garnered the attention of researchers because of its low production cost and customized synthesis.^12^ Compared to conventional binders such as silicones and organic resins, transparent glasses are more suitable for embedding phosphors as they prevent thermal degradation of phosphors due to low sintering temperature. A number of glass systems such as silicates, borates, phosphates, tellurites, and oxy-fluorides have been found suitable for synthesizing PIG structures.^12^ Also, unlike ceramic phosphor plate (CPP) or phosphor glass ceramic (PGC), the PIG structure uses a small amount of phosphor, which lowers the production cost. On the other hand, the PIG structure can be synthesized from a variety of phosphors depending on the type of transparent glass. Ever since its invention, PIG materials involving downconverting phosphors have shown promising results in high-power LEDs.^12–14^ Also, Zeng *et al.* have reported lifetime based optical temperature sensing using the Sm^2+^ doped SrB_4_O_7_:TeO_2_–ZnO PIG structure.^15^

But it is surprising that despite showing such commendable results with downconverting phosphors, reports on upconverting phosphor-based PIG structures are not found to be available to the best of our information. This knowledge gap serves as a basis of the present study, in which the optical thermometry and thermal quenching of luminescence in the Er^3+^/Yb^3+^ codoped NaGd(WO_4_)_2_:TeO_2_–WO_3_–ZnO–TiO_2_ (TWZTi) PIG structure have been studied extensively using 980 nm excitation. NaGd(WO_4_)_2_ is an A^+^B^3+^(WO_4_)_2_ alkali rare-earth double tungstate type host having a monoclinic or tetragonal system and scheelite like structure, where A^+^ = alkali ions (Li^+^, Na^+^, K^+^, Rb^+^ and Cs^+^) and B^3+^ = Y^3+^ and RE^3+^. In this type of structure, the RE and alkali ion occupy the same site, thereby increasing the structural disorder.^16–19^ Moreover, the NaGd(WO_4_)_2_ host matrix having relatively low phonon frequency is more stable and environment friendly in comparison to hosts like fluorides, sulfides, *etc.*^20^ This host has been selected due to its potential in the field of luminescence.^21,22^ On the other hand, TWZTi is a heavy-metal oxide based transparent glass, in which WO_3_, ZnO and TiO_2_ act as network modifiers. The addition of WO_3_ is well-known for enhancing the thermal stability of glass by forming strong Te^__^O^__^W linkages.^23^ The addition of ZnO will increase the density of the glass and decrease its optical bandgap, thereby increasing its refractive index as well. The last modifier TiO_2_ enhances the chemical durability of the glass. Moreover, the addition of TiO_2_ also decreases the optical bandgap of the glass, causing an increase in its refractive index.^24,25^

The current work consists of synthesis of Er^3+^/Yb^3+^:NaGd(WO_4_)_2_ phosphors and structural and optical studies such as XRD, FESEM, FTIR, Raman, UV-vis spectroscopy, UC emission, *etc.* The synthesized phosphors have also been utilized for anticounterfeiting purposes. The thermal stability of the prepared phosphors has been confirmed using the Arrhenius equation. Based on the observations, PIG using (TWZTi) glass has been developed at varying concentrations of optimized phosphors and made applicable for upconversion-based temperature sensing application for the first time. Also, the temperature dependent characteristics of the developed PIGs have been compared to those of the optimized phosphors and glass.

## 2. Material synthesis and characterization

### 2.1 Synthesis of phosphors

The Er^3+^/Yb^3+^:NaGd(WO_4_)_2_ phosphors have been synthesized by the standard solid-state reaction method. The precursors Na_2_CO_3_ (RANKEM > 99.5%), Gd_2_O_3_ (CDH, >99.9%), WO_3_ (CDH, 99.0%), Er_2_O_3_ (CDH, 99.99%) and Yb_2_O_3_ (Sigma-Aldrich, 99.9%) were used in the preparation of the phosphors according to the following chemical expressions:Na_2_CO_3_ + Gd_2_O_3_ + 4WO_3_ → 2NaGd(WO_4_)_2_ + CO_2_(100 − *x*)NaGd(WO_4_)_2_ + *x*Er_2_O_3_where *x* = 0.1, 0.3, 0.5 and 0.7 mol%.(100 − *x* − *y*)NaGd(WO_4_)_2_ + *x*Er_2_O_3_ + *y*Yb_2_O_3_where *x* = 0.5; *y* = 1.0, 3.0, 5.0 and 7.0 mol%.

The mixture was brought to a stoichiometric ratio and pulverized using acetone in a mortar and pestle for 90 minutes. The mixed reagents were maintained at 1000 °C for three hours to facilitate high temperature synthesis in a muffle furnace. After naturally cooling to ambient temperature, the powder phosphors were out of the way for further investigations.

### 2.2 Preparation of glass

As reported,^26,27^ a TeO_2_-based glass system with specifications such as a low phonon energy of 800 cm^−1^, refractive index of 1.97–2.14 and melting temperature of 800 °C has been selected. The bare glass with the 70TeO_2_ + 15WO_3_ + 10ZnO + 5TiO_2_ (TWZTi) molar composition has been prepared with the melt-quenching technique. In this procedure, the high purity (in mol%) powder forms of the glass precursors TeO_2_, WO_3_, ZnO and TiO_2_ were weighed to yield 4 g of glass compositions, and the mixture was then ground in an agate mortar for two hours to obtain a fine and uniform mixture. The raw mixed material compositions were put into alumina crucibles and then heated to a temperature of 900 °C in a high-temperature electric furnace until the entire mixture became a transparent liquid. By pouring the obtained melt into a pre-heated brass mould and covering it with a hot flat brass plate, the melt was quickly quenched. A transparent glass of ∼3 mm thickness was obtained, as shown in Fig. 2(f), and used for PIG development. Also, a separate optimized Er^3+^/Yb^3+^:TWZTi glass was synthesized for comparative study. The dopant concentration was kept the same as that of phosphors.

### 2.3 Preparation of PIG

The obtained undoped TWZTi glass was crushed into powders (glass frits) and mixed with optimized Er^3+^/Yb^3+^:NaGd(WO_4_)_2_ phosphors. The phosphors in glass with the concentration of 1.0, 5.0, 10.0 and 20.0 weight% were named PIG1, PIG2, PIG3 and PIG4, respectively. The mixture powder of 0.7 g was used to make pellets and sintered at 550 °C for 45 min. The as-prepared PIGs were used for the photoluminescence study.

### 2.4 Characterization

To determine the crystal formation and lattice parameters, the optimized Er^3+^/Yb^3+^:NaGd(WO_4_)_2_ phosphors were analyzed using an X-ray diffractometer in the 10° ≤ 2*θ* ≤ 80° range. FESEM consisting of an airlock compartment has been used for morphological analysis. The vibrational bands have been detected with FTIR. XPS confirms the valence states and binding energies of the elements in phosphors. Diffuse reflectance spectra (DRS) have been monitored in the UV-Vis-NIR region. The continuous wave (CW) laser source has been used to achieve the frequency UC. The monochromator used for this purpose consists of a triple grating and a photomultiplier tube. Thermal stability experiments have been carried out using a multimeter, thermocouple, and a small heater that is maintained by altering the voltage. Calculations of CIE coordinates have been performed using GoCIE software.

## 3. Results and discussion

### 3.1 Structural characterization

**Fig. 1:**
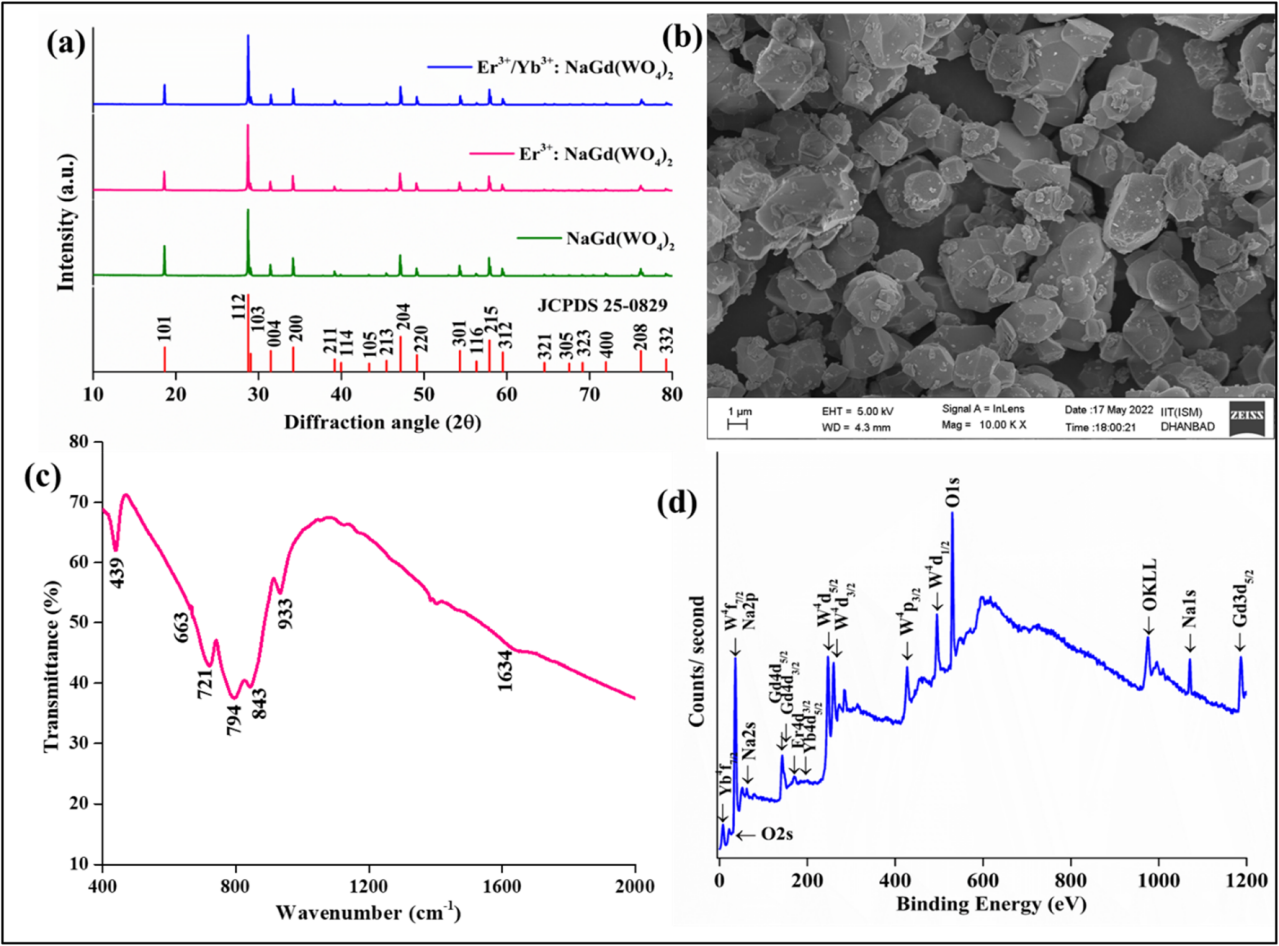
(a) XRD spectra of NaGd(WO_4_)_2_, Er^3+^:NaGd(WO_4_)_2_ and Er^3+^/Yb^3+^:NaGd(WO_4_)_2_ phosphors, (b) FESEM of optimized Er^3+^/Yb^3+^:NaGd(WO_4_)_2_ phosphors, (c) FTIR spectrum of the NaGd(WO_4_)_2_ host and (d) XPS of Er^3+^/Yb^3+^:NaGd(WO_4_)_2_ phosphors.

The crystal formation of the developed Er^3+^/Yb^3+^:NaGd(WO_4_)_2_ phosphors has been confirmed with the XRD pattern {Fig. 1(a)}. The peak positions coincide with the JCPDS file 25-0829 with no impurity phase. The NaGd(WO_4_)_2_ has a scheelite-like tetragonal structure with the *I*4_1_/*a* space group and cell parameters of *a* = *b* = 5.22 Å, *c* = 11.32 Å, *α* = *β* = γ = 90° and volume = 309.21 Å^3^. The XRD peaks of the phosphors correspond to (101), (112), (103), (004), (200), (211), (114), (105), (213), (204), (220), (301), (116), (215), (312), (321), (305), (323), (400), (208) and (332) (*hkl*) planes. The crystallite size (*D*) can be given by the Debye–Scherrer equation,^28^1
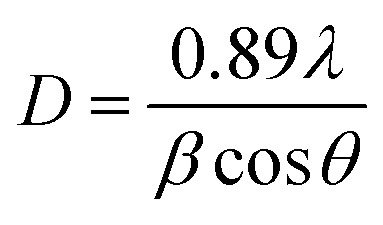
where ‘*λ*’ denotes the wavelength of X-ray used (*λ* = 1.5406 Å), ‘*β*’ signifies the full width at half maximum and ‘*θ*’ represents the Bragg's diffraction angle. The crystallite sizes of ∼74.65 nm, ∼67.67 nm and ∼59.70 nm of the NaGd(WO_4_)_2_, Er^3+^:NaGd(WO_4_)_2_ and Er^3+^/Yb^3+^:NaGd(WO_4_)_2_ phosphors have been calculated, respectively. Again, using the relation between the radius of the host cation ‘*R*_m_(CN)’ and doped ion ‘*R*_d_(CN)’, the acceptable percent of doping (*D*_r_) can be confirmed as^29^2
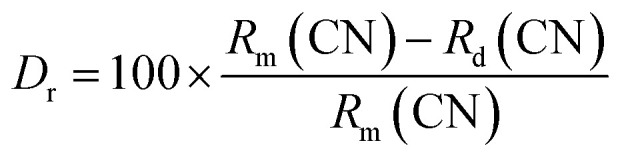
where the ionic radii of Na^+^, Gd^3+^, W^6+^, Er^3+^ and Yb^3+^ are 1.02 Å, 0.93 Å, 0.60 Å, 0.89 Å and 0.86 Å in VI coordination, respectively.^30^ The value of *D*_r_ was found to be 4% for Er^3+^ and 7% for Yb^3+^ doping in Gd^3+^ sites of the NaGd(WO_4_)_2_ crystal, which are in agreement with the fact *D*_r_ < 30%. The substitution of rare earths with a similar ionic radius and the same ionic charge does not disturb the crystal structure.

The FESEM image of prepared Er^3+^/Yb^3+^:NaGd(WO_4_)_2_ phosphors is shown in Fig. 1(b) on the 1 μm scale with almost circular shape particles of various sizes.

The vibrational bands at ∼439 cm^−1^, ∼663 cm^−1^, ∼721 cm^−1^, ∼794 cm^−1^, ∼843 cm^−1^, ∼933 cm^−1^ and ∼1634 cm^−1^ have been detected in the FTIR spectrum of the host NaGd(WO_4_)_2_ {Fig. 1(c)}. The broad peak at ∼1634 cm^−1^ is due to the stretching vibration of the hydroxyl (-OH) group. The peak at ∼933 cm^−1^ corresponds to W–O asymmetric stretching vibrations.^31^ The band at ∼843 cm^−1^ is resulted from O–W–O stretching of the WO_4_ tetrahedron, which confirms that the prepared phosphors are AWO_4_ type scheelite oxides and S_4_ site symmetry for the (WO_4_)_2_ groups.^31,32^ The vibrational energy of phosphors represents the maximum possible non-radiative transitions during excitation and emission processes. The other stretching vibrations of the WO_4_ tetrahedron have been observed at 663 cm^−1^, 721 cm^−1^ and 794 cm^−1^. The 439 cm^−1^ peak is related to the bending mode of the WO_4_ tetrahedron.^32^ The detected FTIR bands in the 400–2000 cm^−1^ range are well matched with those of the double tungstate structure.^31,32^

Fig. 1(d) displays the XPS spectrum of Er^3+^/Yb^3+^:NaGd(WO_4_)_2_ phosphors. The spectrum confirms the elements present in the prepared phosphors. The calibration has been performed with respect to the C 1s (∼284 eV) peak. The XPS peaks of oxygen are observed at ∼21 eV (O 2s) and ∼530 eV (O 1s). One Auger peak at ∼974 eV (OKLL) has also been detected in the XPS spectrum.^33^ The peaks for tungsten are found at ∼35 eV (4f_7/2_), ∼247 eV (4d_5/2_), ∼259 eV (4d_3/2_), ∼426 eV (4p_3/2_) and ∼495 eV (4p_1/2_).^33,34^ The XPS peaks of sodium are detected at ∼35 eV (2p), ∼62 eV (2s) and ∼1071 eV (1s).^34^ The two overlapped peaks of gadolinium are at ∼143 eV and ∼147 eV due to 4d_5/2_ and 4d_5/2_ states.^34,35^ One more peak of Gd has been found at ∼1186 eV (3d_5/2_).^34^ The binding energy peak for Er^3+^ has been found at ∼170.0 eV (4d_3/2_). The ∼7.0 eV and ∼184 eV peaks correspond to 4f_5/2_ and 4d_5/2_ states due to Yb^3+^ ions.^33^

### 3.2 Optical study

**Fig. 2:**
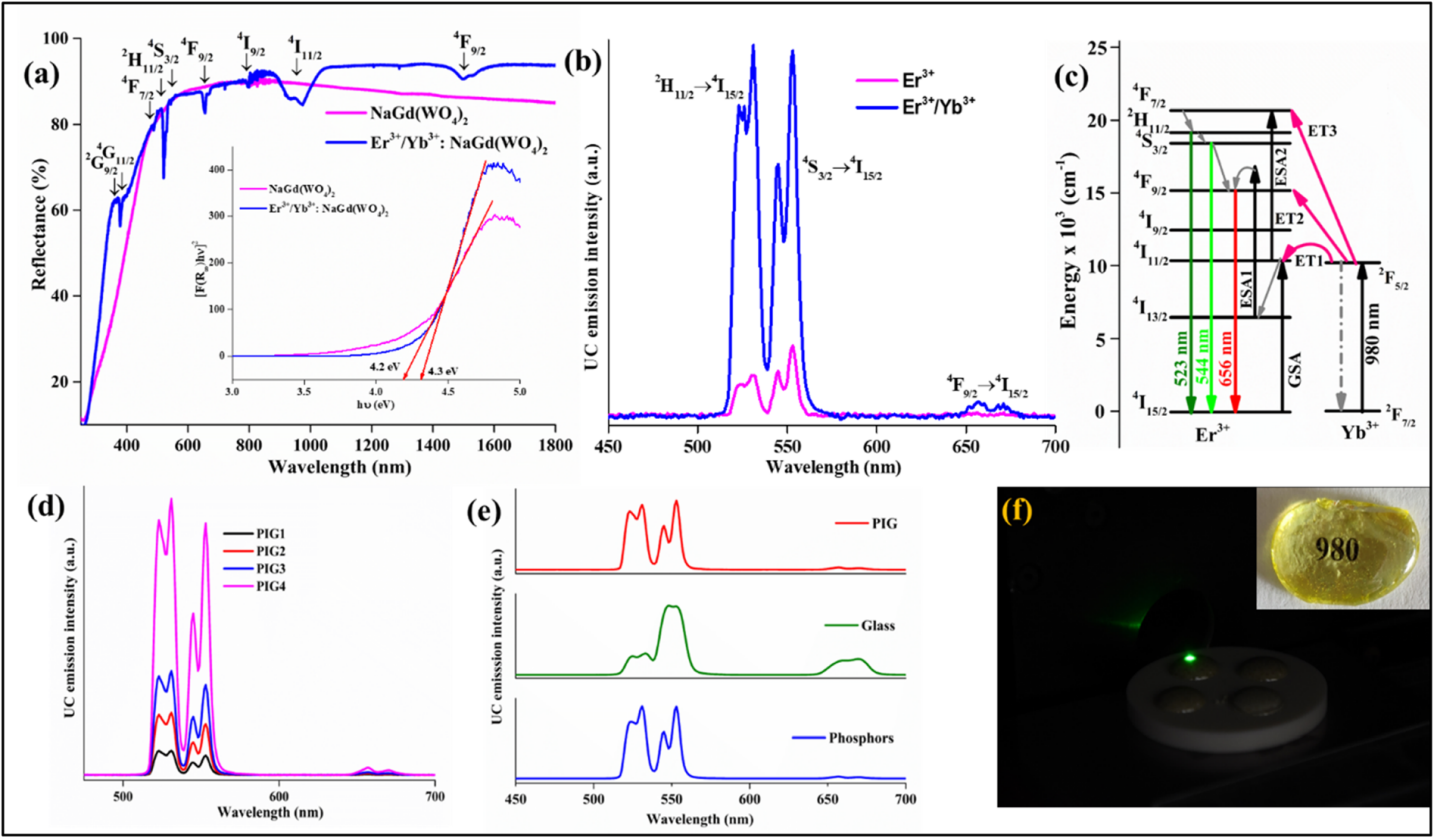
(a) DRS spectra and calculated bandgap of the NaGd(WO_4_)_2_ host and optimized Er^3+^/Yb^3+^:NaGd(WO_4_)_2_ phosphors, (b) UC emission of optimized Er^3+^ and Er^3+^/Yb^3+^:NaGd(WO_4_)_2_ phosphors, (c) energy level diagram of the Er^3+^/Yb^3+^ system, (d) UC emission from PIGs with variation of phosphor contents (1.0, 5.0, 10.0 and 20.0 weight%), (e) comparative UC emission spectra for phosphors, glass and PIG and (f) developed PIGs on 980 nm excitation; the inset shows the transparency of the as-prepared undoped glass.

The UV-vis spectra of the NaGd(WO_4_)_2_ and optimized 0.5 mol% Er^3+^/3.0 mol% Yb^3+^:NaGd(WO_4_)_2_ phosphors have been recorded in the 200–1800 nm wavelength range {Fig. 2(a)}. The spectra were calibrated with the standard BaSO_4_ powder. The host NaGd(WO_4_)_2_ does not contain any absorption peak. The Er^3+^/Yb^3+^:NaGd(WO_4_)_2_ phosphor consists of various absorption peaks at ∼366 nm, ∼378 nm, ∼488 nm, ∼520 nm, ∼529 nm, ∼544 nm, ∼551 nm, ∼655 nm, ∼677 nm, ∼799 nm, ∼974 nm and ∼1498 nm. The respective transitions originated from the ground state (^4^I_15/2_) of Er^3+^ ions to the ^2^G_9/2_, ^4^G_11/2_, ^4^F_7/2_, ^2^H_11/2 (I)_, ^2^H_11/2 (II)_, ^4^S_3/2 (I)_, ^4^S_3/2 (II)_, ^4^F_9/2_, ^4^I_9/2_, ^4^I_11/2_ and ^4^I_13/2_ excited states. The intense peak at 974 nm has been observed due to the overlapping transitions from the Yb^3+^ and Er^3+^ ions. Further, the DRS spectra can be used to find the bandgap of the developed phosphors using the following Kubelka–Munk function *F*(*R*_∞_) and Tauc relationship by^36^

3 [*F*(*R*_∞_)*hν*] = *C*(*hν* − *E*_g_)^*n*^

4 *F*(*R*)= (1 − *R*)^2^/2*R* = *K*/*S*

where *F*(*R*_∞_) represents the reflectance of the infinitely thick sample with respect to the reference at each wavelength, ‘*C*’ is a constant, ‘*hν*’ signifies the photon energy, ‘*E*_g_’ indicates the bandgap and *n* = ½ for direct bandgap. ‘*R*’ designates the diffuse reflectance of the spectrum, ‘*K*’ signifies the absorption coefficient and ‘*S*’ is the scattering coefficient. The calculated bandgaps for NaGd(WO_4_)_2_ and Er^3+^/Yb^3+^:NaGd(WO_4_)_2_ phosphors are ∼4.2 eV and ∼4.3 eV, respectively, with negligible difference {Fig. 2(a), inset}.

#### Frequency upconversion emission studies

The UC emission spectra of the prepared phosphors have been recorded at room temperature upon 980 nm excitation over the 450–700 nm {Fig. 2(b)} region and the optimal concentrations of Er^3+^ and Yb^3+^ have been found to be 0.5 and 3.0 mol%, respectively. The 0.5 mol% Er^3+^:NaGd(WO_4_)_2_ phosphors contain green emission peaks at ∼523 nm and ∼544 nm, corresponding to the ^2^H_11/2_ (H) → ^4^I_15/2_ and ^4^S_3/2_ (S) → ^4^I_15/2_ transitions, and a red emission peak at ∼656 nm due to the ^4^F_9/2_ → ^4^I_15/2_ transition. Beyond 0.5 mol% concentration, quenching of luminescence intensity has been observed in the prepared phosphors. To understand the reason behind the quenching, the critical distance has been calculated using Blesse's equation.^29^ The critical distance represents the value up to which the energy transfer between two Er^3+^ ions can take place. The obtained value of critical distance is ∼6.6 Å, which is greater than 5 Å, indicating that the quenching has occurred due to multipolar interaction.^29^ Further, to get an insight into the type of multipolar interaction, Dexter and Van Uitert's relationship has been used.^29^ The obtained slope value (∼8) indicates that the multipolar interaction between the Er^3+^ ions is electric dipole–quadrupole in nature. This interaction is mainly responsible for the concentration quenching in the doped NaGd(WO_4_)_2_ phosphors. Codoping with Yb^3+^ ions enhances the luminescence intensity by ∼7 times by transferring the excitation energy from the Yb^3+^ to Er^3+^ ions.

The pump-power dependent UC emission spectra of the optimized Er^3+^ and Er^3+^/Yb^3+^:NaGd(WO_4_)_2_ phosphors have been recorded by varying the pump power density from 1.36 to 75.9 W cm^−2^. With the increase in pump power density, the UC emission intensity increases. However, in Er^3+^/Yb^3+^:NaGd(WO_4_)_2_ phosphors, the UC emission intensity beyond 66.9 W cm^−2^ decreases. This decrease can be attributed to the thermal quenching effect generated in the samples due to the exposure to 980 nm CW laser radiation.^37^ The UC emission intensity (*I*) and pump power (*p*) are related by the expression *I* ∝ *p*^*n*^, where ‘*n*’ is the pump photons necessary for populating energy levels. The ‘*n*’ values for H and S levels in the case of 0.5 mol% Er^3+^ doped NaGd(WO_4_)_2_ phosphors are ∼1.7 and ∼1.5, respectively. The deviation of the slope value from two is due to the involvement of ESA and cross relaxation processes.^19^ In the case of 0.5 Er^3+^/3.0 Yb^3+^ doped NaGd(WO_4_)_2_ phosphors the ‘n’ values are ∼1.2 and ∼1.0. The decrease in the slope value from the standard value (∼2) is due to the competition between the linear decay and the upconversion processes. This competition has been theoretically explained in detail on the basis of rate-law equations.^37,38^

Further, PIG has been developed with varying contents of optimized phosphors. With the increase of phosphor content, the UC emission intensity increases. With the increase in pump power density as well, the UC emission intensity increases and the calculated slope value ‘n’ for PIG is ∼1.4 and ∼1.2 for H and S levels, respectively. However, the less intensity of PIG compared to phosphors is mainly due to the smaller composition (20%) of phosphors in the glass frits {Fig. 2(d)}. The UC emission spectra of developed phosphors, PIG and glass have been recorded for comparative study {Fig. 2(e)}. The PIG contains the characteristic peak shape of the developed phosphors. In the optimized glass, the intensity of the red emission band has increased, but the stark levels in green bands are not visible. The UC emission from the developed PIG upon 980 nm excitation is shown in Fig. 2(f).

The UC mechanism upon 980 nm excitation in the Er^3+^, Er^3+^/Yb^3+^ system has been explained with the energy level diagram {Fig. 2(c)}. In Er^3+^ doped NaGd(WO_4_)_2_ phosphors, the energy levels are populated by GSA/ESA processes. By absorbing 980 nm photons, Er^3+^ ions are excited from the ground state (^4^I_15/2_) to the excited state (^4^I_11/2_) through the GSA process. From there, some of the ions decay *via* a non-radiative process to the ^4^I_13/2_ level and then again excited to the ^4^F_9/2_ level *via* the ESA1 process. The radiative transition from the ^4^F_9/2_ level gives red emission at 656 nm. Apart from this, few ions in the ^4^I_11/2_ levels get excited to the ^4^F_7/2_ level by sequential absorption *via* the ESA2 process. From the ^4^F_7/2_ level the ions relax further to the ^2^H_11/2_ and ^4^S_3/2_ levels. The radiative transitions from the ^2^H_11/2_ and ^4^S_3/2_ levels result in green emissions at 523 nm and 544 nm, respectively. The codoping with Yb^3+^ ions enhances the UC emission intensity associated with the following energy transfer processes:ET1: ^2^F_5/2_ (Yb^3+^) + ^4^I_15/2_ (Er^3+^) → ^2^F_7/2_ (Yb^3+^) + ^4^I_11/2_ (Er^3+^)ET2: ^2^F_5/2_ (Yb^3+^) + ^4^I_13/2_ (Er^3+^) → ^2^F_7/2_ (Yb^3+^) + ^4^F_9/2_ (Er^3+^)ET3: ^2^F_5/2_ (Yb^3+^) + ^4^I_11/2_ (Er^3+^) → ^2^F_7/2_ (Yb^3+^) + ^4^F_7/2_ (Er^3+^), ^4^F_7/2_ (Er^3+^) → NRR → ^2^H_11/2_, ^4^S_3/2_.

#### Photometric characterization and security ink application

**Fig. 3:**
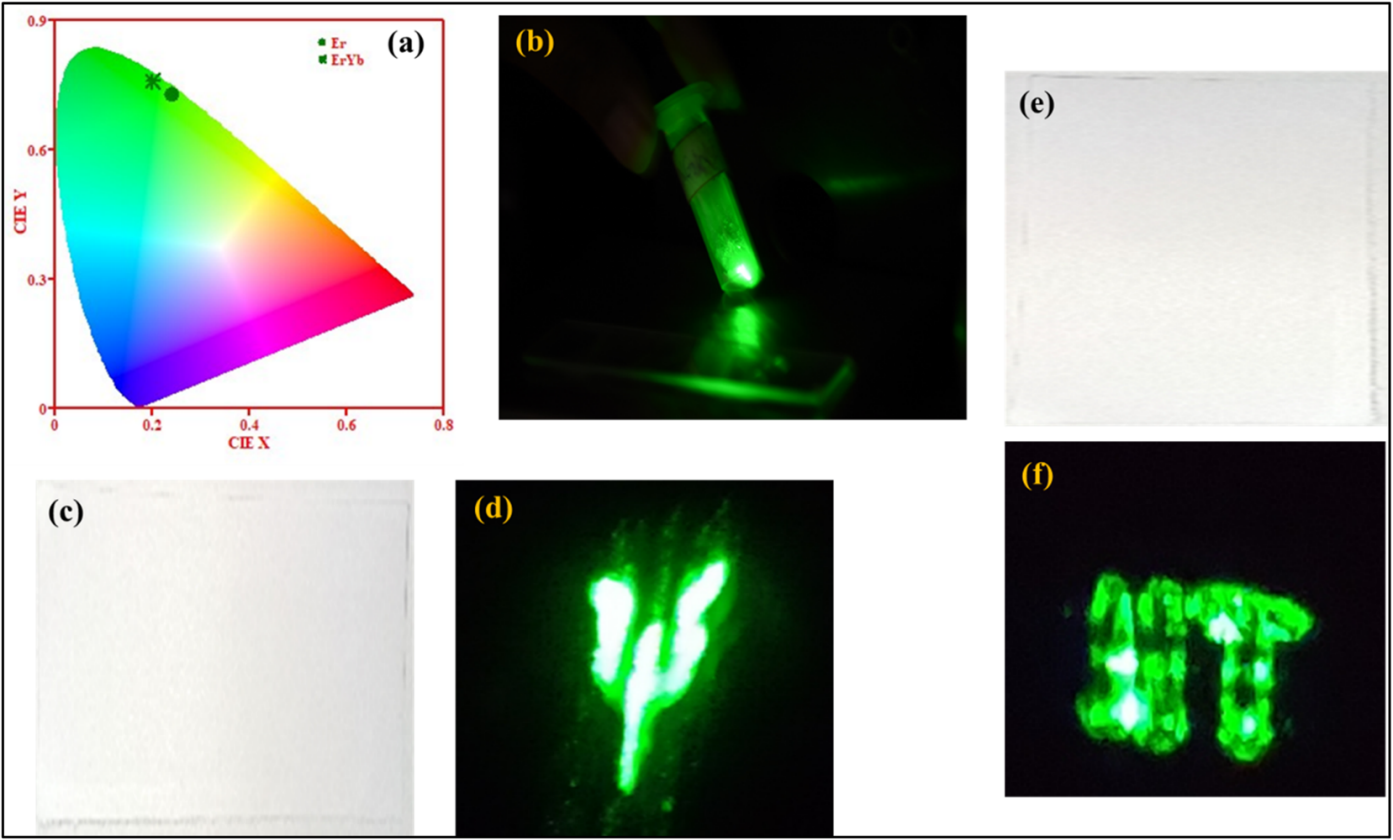
(a) CIE diagram of Er^3+^:NaGd(WO_4_)_2_ and Er^3+^/Yb^3+^:NaGd(WO_4_)_2_ phosphors, (b) green emission from optimized Er^3+^/Yb^3+^:NaGd(WO_4_)_2_ phosphors, and (c) and (e) the letter ‘ψ’ and letters ‘IIT’ upon natural day light illumination, and (d) and (f) upon 980 nm excitation.

The colour emission from the phosphors has been visualized with CIE coordinates. The color coordinates for Er^3+^:NaGd(WO_4_)_2_ and Er^3+^/Yb^3+^:NaGd(WO_4_)_2_ phosphors were calculated to be (0.23, 0.73) and (0.20, 0.76), respectively {Fig. 3(a)}. The CIE coordinates shifted towards the greenish region with the incorporation of Yb^3+^ ions in the Er^3+^:NaGd(WO_4_)_2_ phosphors. The UC emission from codoped phosphors upon 980 nm excitation is shown in Fig. 3(b). The developed phosphors have been applied for making security inks. The letters ‘Ψ’ and ‘IIT’ have been written on white paper by dissolving phosphors into ethanol {Fig. 3(c and e)}. The letters were invisible under natural day light illumination, but upon 980 nm excitation the letters became visible {Fig. 3(d and f)}. In this way, the present upconverting phosphors can protect from counterfeiting threats by keeping confidential documents and valuable products secured.

#### Temperature sensing

The thermally coupled energy levels (TCLs) present in the Er^3+^ can be explored for temperature sensing application using the fluorescence intensity ratio (FIR) approach. The temperature dependent UC emission spectra of the Er^3+^/Yb^3+^:NaGd(WO_4_)_2_ phosphors (296–623 K), glass (296–623 K) and PIG (298–523 K) have been recorded. In both cases, the population at the H level increases up to 403 K and then decreases with respect to temperature. The TCL-based FIR technique can be applied to the UC peaks at 523 nm (^2^H_11/2_) and 544 nm (^4^S_3/2_) of Er^3+^ ions, as the energy difference between the ^2^H_11/2_ and ^4^S_3/2_ levels is ∼738 cm^−1^. The effect of rising temperature on the TCLs (H and S), *i.e.*, temperature-induced population redistribution ability (PRA), can be analyzed with the expression^36,39^5
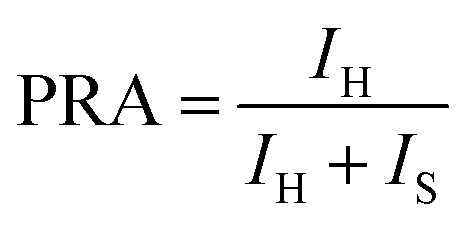


**Fig. 4:**
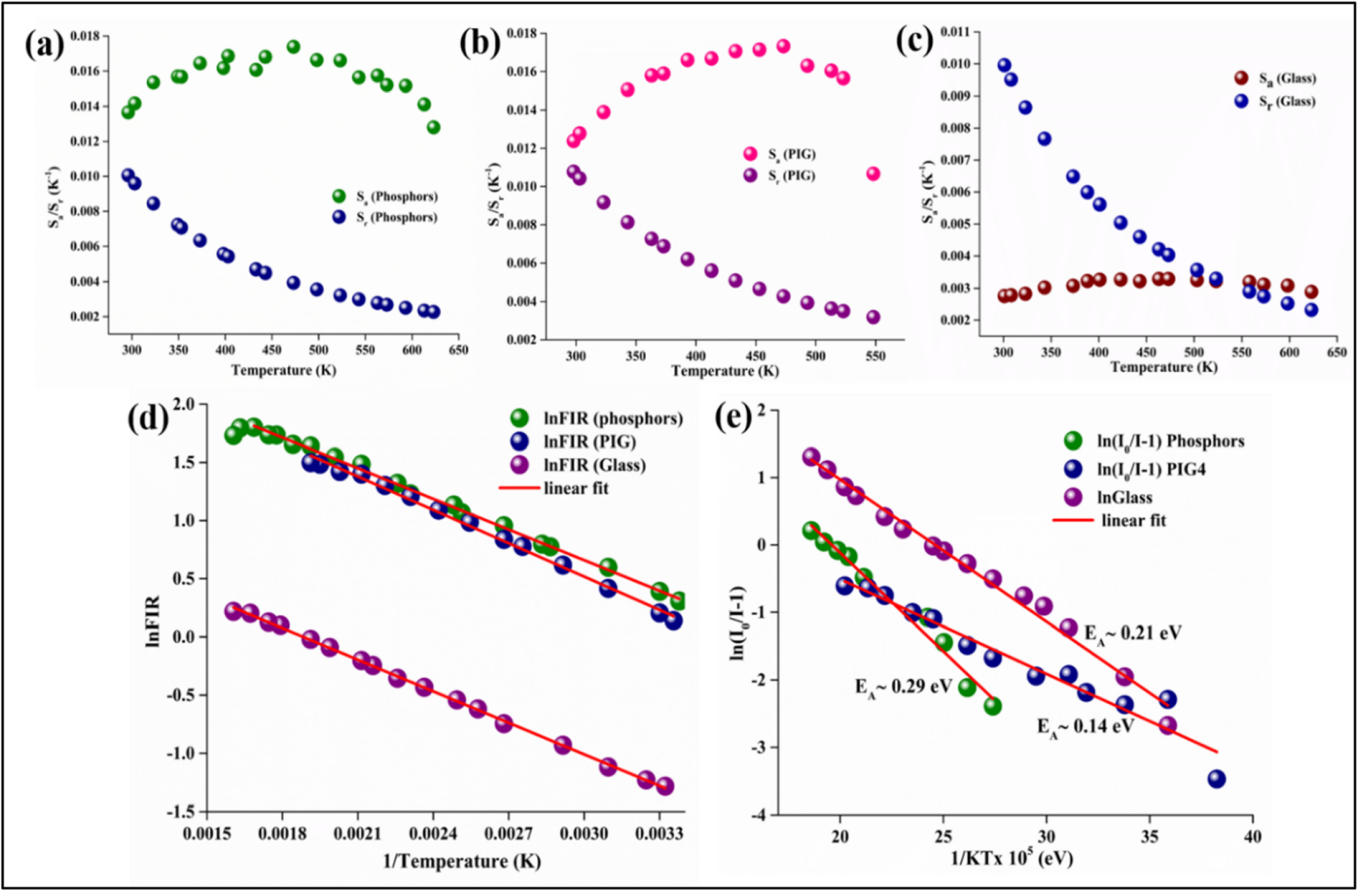
Absolute and relative sensitivity of Er^3+^/Yb^3+^:NaGd(WO_4_)_2_ (a) phosphors, (b) PIG, and (c) glass, (d) plot of ln FIR *vs.* 1/*T* (K) and (e) activation energy of phosphors, PIG and glass.

PRA for phosphors varies from 0.58 to 0.85 (296–623 K), from 0.53 to 0.82 (296–523 K) for PIG and 0.22 to 0.55 (301–623 K) for glass. The population of TCLs follows the Boltzmann distribution and FIR is given by the relation^36,39^6
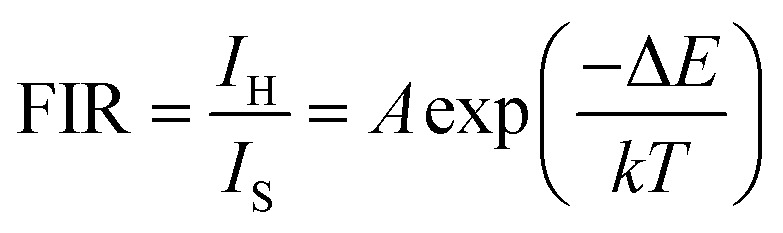
where *I*_H_ and *I*_S_ are the integrated intensities of thermally coupled green bands, ‘*A*’ is the proportionality constant, Δ*E* is the energy difference between these two levels and *k* is Boltzmann's constant (0.695 K^−1^ cm^−1^). Δ*E* between the ^2^H_11/2_ and ^4^S_3/2_ levels can be determined by linear fitting {Fig. 4(d)}. For phosphors, the obtained Δ*E* value is ∼612.31 cm^−1^, for PIG it is ∼956.85 cm^−1^ and for glass it is ∼902 cm^−1^. Further, error ‘*δ*’, the discrepancy between the fitted energy difference (Δ*E*) and the experimental energy difference (Δ*E*_0_), can be used to assess the accuracy of temperature sensing using the expression^39^7
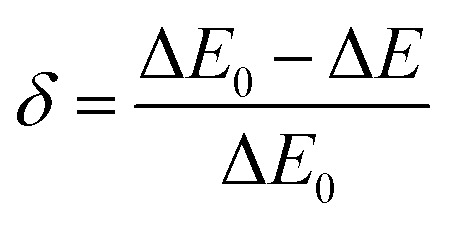


The calculated errors for phosphors, PIG and glass come out to be 0.20, 0.10 and 0.15, respectively. The obtained value of *δ* is small and is minimum in the case of PIG. Thus, the weak energy transfer between the two thermally coupled levels and other levels can be neglected.^41^

The obtained Δ*E* can be used to find the sensitivities, *i.e.*, how often a sensor can detect even the smallest temperature changes. The relative (*S*_r_) and absolute (*S*_a_) sensitivities can be estimated by the following expressions^21,42^8
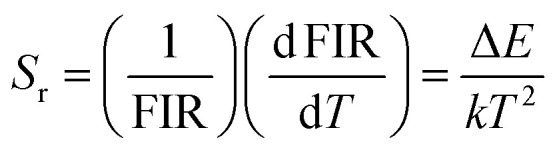
9
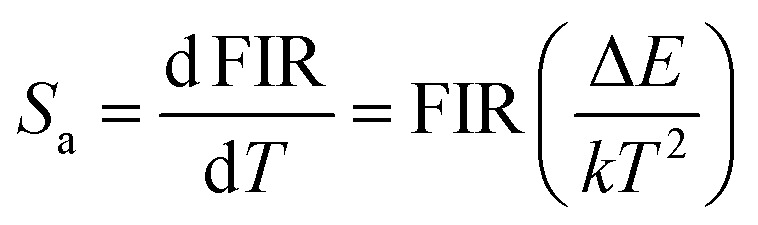


The graph for *S*_r_ and *S*_a_ demonstrates that the absolute sensitivity rises with temperature, reaching a maximum sensitivity, and then decreases {Fig. 4(a)–(c)}. For both phosphors and PIG, the *S*_a_ is 17.3 × 10^−3^ K^−1^ @ 473 K. However, the relative sensitivity is found to be 10.0 × 10^−3^ K^−1^ @ 296 K for phosphors and a maximum of 10.7 × 10^−3^ K^−1^ @ 298 K for the PIG. Although there is a difference in Δ*E* value for phosphors and PIG, no detectable change in the sensitivities has been observed. The low intensity observed for PIG does not affect the sensitivity. Thus, both phosphors and PIG can be used for sensor development. Besides, for Er^3+^/Yb^3+^:glass the values of *S*_a_ and *S*_r_ are found to be 3.29 × 10^−3^ K^−1^ @ 473 K and 9.96 × 10^−3^ K^−1^ @ 301 K, respectively.

The sensitivities for different optical temperature sensing materials are listed in Table 1. Further, the temperature where a sensor performs best, *i.e.*, maximum sensitivity *S*_max_ and the temperature *T*_max_ can be obtained by putting d*S*_a_/d*T* = 0 as^38,43^10
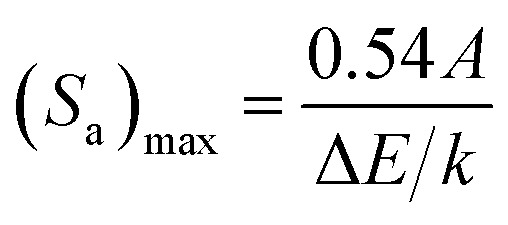
11
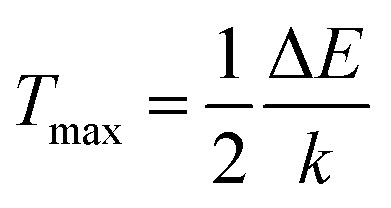


**Table 1:** Comparison of sensitivities in different Er^3+^/Yb^3+^ doped tungstate materials using the FIR technique in ^2^H_11/2_ and ^4^S_3/2_ levels

Phosphors/glass/phosphors in glass	Temperature range (K)	*S*_a_ (×10^−3^ K^−1^)	*S*_r_ (×10^−3^ K^−1^)	Reference
Er^3+^/Yb^3+^:BaWO_4_ phosphors	293–393	10.7 @ 293 K	14.7 @ 393 K	44
Er^3+^/Yb^3+^:MgWO_4_ phosphors	302–623	14.4 @ 473 K	9.72 @ 302 K	45
Er^3+^/Yb^3+^:SrWO_4_ phosphors	300–518	14.9 @ 403 K	5.3 @ 403 K	46
Er^3+^/Yb^3+^:Ca_2_MgWO_6_ phosphors	303–573	8.2 @ 453 K	9.2 @ 303 K	47
Er^3+^/Yb^3+^:GdBiW_2_O_9_ phosphors	303–498	17.4 @ 450 K	1.2 @ 303 K	48
Er^3+^/Yb^3+^:NaBi(WO_4_)_2_ phosphors	298–373	12.7 @ 373 K	12.4 @ 298 K	49
Er^3+^/Yb^3+^:NaY(WO_4_)_2_ phosphors	298–573	14.6 @ 523 K	3.9 @ 523 K	50
Er^3+^/Yb^3+^:NaGd(WO_4_)_2_ glass	303–573	12.0 @ 542 K	1.1 @ 303 K	51
Er^3+^/Yb^3+^:NaGd(WO_4_)_2_ phosphors	296–623	17.3 @ 473 K	10.0 @ 296 K	Present work
Er^3+^/Yb^3+^:NaGd(WO_4_)_2_ PIG	298–523	17.3 @ 473 K	10.7 @ 298 K	Present work
Er^3+^/Yb^3+^:NaGd(WO_4_)_2_ glass	301–623	3.29 @ 473 K	9.96 @ 301 K	Present work

*S*_max_ and *T*_max_ for Er^3+^/Yb^3+^:NaGd(WO_4_)_2_ phosphors have been determined to be 16.6 × 10^−3^ K^−1^ and 440 K, respectively. These values are closely in agreement with the outcome of the experimental calculation using eqn (8) and (9). Eqn (10) states that for higher sensitivity, a larger pre-exponential factor A and a smaller energy gap between the two levels are needed, whereas eqn (11) demonstrates that for higher *T*_max_, a large energy gap between the two levels is required.

Additionally, the least amount of temperature variation felt by an optical sensor, or the thermal resolution (uncertainty; *T*) in measurements of sensing parameters, can be determined using the following equation^40^12
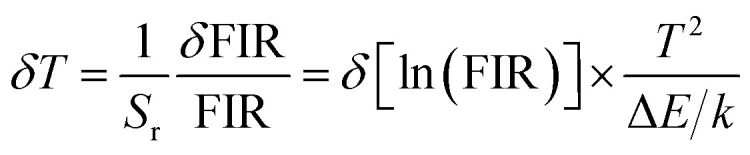
where *δ* [ln(FIR)] is the standard deviation of the linear fit to ln(FIR). The obtained value shows that the thermal resolution at room temperature has been improved from 0.50 and 0.49 to 0.42 for glass and phosphors to PIG.

#### Thermal stability

Highly efficient phosphors in terms of thermal stability are required for the applications of solid-state lighting and temperature sensors. As the chromaticity, color shift, color rendering index, service life, absorption intensity and transition probability can be affected by the increase in temperature, the thermal stability of phosphors becomes a significant parameter.^21^ The quenching intensity and the spectral shape with rising temperature are two factors that can be used to assess thermal stability. Therefore, temperature dependent UC emission of the Er^3+^/Yb^3+^:NaGd(WO_4_)_2_ phosphors (296–623 K), PIG (300–573 K) and glass (301–623 K) has been recorded. No change in the shape of the UC emission spectra has been observed. However, thermal quenching with the increase in temperature has been noticed, which is a major challenge of photoluminescence of any material. In actuality, at high temperatures, energy transmission becomes active due to lattice vibrations of the host to rare earth ions. Unexpectedly, the green band of the as-prepared phosphors exhibits a very low luminescence quenching behavior. Their UC emission intensity retains 92% when measured at 423 K of the initial intensity measured at 296 K and it is found to be 84% for the PIG and only 62% for glass. The behavior of the temperature dependent UC emission intensity has also been observed on further increasing the temperature and does not diminish even at 573 K. The UC intensity retains 54%, 65% and 30% of the initial intensity at 573 K for the phosphors, PIG and glass, respectively. Here, an improvement of 10% intensity compared to that of phosphors can be noticed with the PIG, which is 30% compared to that of glass. Thus, PIG can be an effective method to improve thermal quenching at higher temperatures. The temperature scale without much intensity loss has been enlarged using the phosphors as well as PIG (Table 2). The comparative study of the retained luminescence intensity with different phosphors shows that the initial intensity of the present Er^3+^/Yb^3+^:NaGd(WO_4_)_2_ phosphor reduces by only 8% at 423 K, thereby exhibiting excellent thermal stability.

**Table 2:** The percentage of UC emission intensity retained at 423 K due to thermal quenching has been compared to other reported works

Phosphors/glass/phosphors in glass	Retained intensity at 423 K		References
RaBa_2_(PO_3_)_5_:Eu^2+^ phosphors	89.5%		52
CaMgSi_2_O_6_:Cr^3+^ phosphors	88.4%		53
NaGd(MoO_4_)_2_:Er^3+^–Yb^3+^ phosphors	87%		40
Sr_3_LiTaO_6_:Mn^4+^ phosphors	76.7%		54
Bi_4_Ti_3_O_12_:Er^3+^–Yb^3+^–Al^3+^ phosphors	73%		38
YPO_4_:Ho^3+^–Tm^3+^–Yb^3+^ phosphors	56%		55
YAG:Ce^3+^ phosphor-in SiO_2_–Na_2_O–B_2_O_3_–CaO glass	70% at 503 K		56
SrGa_2_S_4_:Eu^2+^ phosphor-in SiO_2_–B_2_O_3_–ZnO–Al_2_O_3_–K_2_O glass	87% at 423 K		57
YAG:Ce^3+^ phosphor-in SiO_2_–B_2_O_3_–RO (R = Ba, Zn) glass	93% at 473 K		58
YAG:Ce^3+^ phosphor-in SiO_2_–Al_2_O_3_–B_2_O_3_–ZnO–BaO glass	93.6% at 423 K and 88.6% at 473 K		59
NaGd(WO_4_)_2_: Er^3+^/Yb^3+^ phosphors	92% at 423 K	54% at 573 K	Present phosphors
NaGd(WO_4_)_2_: Er^3+^/Yb^3+^ phosphor-in TeO_2_–WO_3_–ZnO–TiO_2_ glass	84% at 423 K	65% at 573 K	Present PIG
TeO_2_–WO_3_–ZnO–TiO_2_: Er^3+^/Yb^3+^ glass	62% at 423 K	30% at 573 K	Present glass

Again, the activation energy has been determined using the Arrhenius equation,^60,61^13
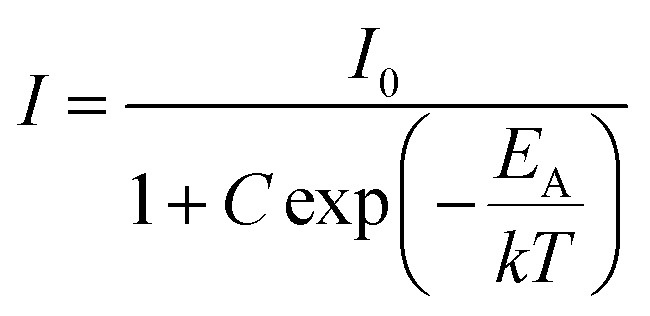
where the luminescence intensities *I*_0_ and *I* are defined at initial temperature *T*_0_ and temperature *T* (K), *E*_A_ stands for the activation energy, ‘*C*’ is a constant and ‘*k*’ signifies the Boltzmann constant (8.629 × 10^−5^ eV K^−1^). The calculated activation energies (*E*_A_) are ∼0.29 eV for the phosphors, ∼0.14 eV for PIG and ∼0.21 eV for the glass {Fig. 4(e)}. The probable non-radiative transition occurring per unit time (*α*) can be estimated by the relation^62^14
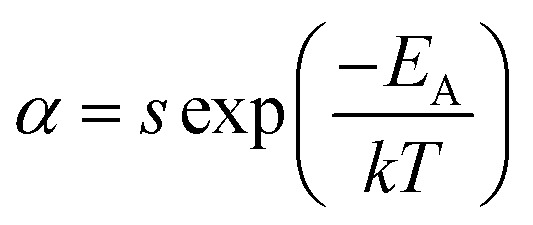
where *s* is a constant. It becomes clear that higher activation leads to less non-radiative transition.

## 4. Conclusion

Er^3+^/Yb^3+^:NaGd(WO_4_)_2_ phosphors have been successfully synthesized and phosphors in glass at various concentrations of phosphors have been developed. The UC emission intensity of Er^3+^ doped NaGd(WO_4_)_2_ phosphors has been enhanced ∼7 times with the addition of Yb^3+^ ions. The prepared phosphors have been made applicable for security purposes. The intensity of the UC emission from PIG rises with the increase in phosphor content. No significant improvement or deterioration has been observed in the absolute and relative sensitivities of PIG as compared to the optimized phosphor. However, the thermal resolution at room temperature has been improved significantly in the case of PIG as compared to the phosphor. Also, at higher temperatures, the luminescence quenching in PIGs has been improved up to 10% and 30% as compared to that of the Er^3+^/Yb^3+^:NaGd(WO_4_)_2_ phosphor and Er^3+^/Yb^3+^:TWZTi glass, respectively. Thus, without much luminescence intensity loss, phosphors and the concept of designing PIG both are applicable for optical temperature sensing and solid-state lighting applications.

## Conflicts of interest

There are no conflicts to declare.

## Supplementary Material

## References

[cit1] Lin S., Chen M., Wang Z., Zhang Y., Yuan R., Liang X., Xiang W., Zhou Y. (2017). Chem. Eng. J..

[cit2] Zhou Z., Zhou N., Xia M., Yokoyama M., Hintzen H. B. (2016). J. Mater. Chem. C.

[cit3] Tamaya T., Ishikawa A., Ogawa T., Tanaka K. (2016). Phys. Rev. Lett..

[cit4] Ying W., Mao Y., Wang X., Guo Y., He H., Ye Z., Lee S. T., Peng X. (2017). ChemSusChem.

[cit5] Zhong J., Chen D., Zhou Y., Wan Z., Ding M., Ji Z. (2016). J. Eur. Ceram. Soc..

[cit6] Hu S., Cao H., Wu X., Zhan S., Wu Q., Tang Z., Liu Y. (2016). J. Nanomater..

[cit7] Kumari A., Soni A. K., Rai V. K. (2016). Mater. Focus.

[cit8] Richards B. S. (2006). Sol. Energy Mater. Sol. Cells.

[cit9] Yang Y., Mi C., Jiao F., Su X., Li X., Liu L., Zhang J., Yu F., Liu Y., Mai Y. (2014). J. Am. Ceram. Soc..

[cit10] Wu S., Ning Y., Chang J., Zhang S. (2013). J. Lumin..

[cit11] Chen B., Wang F. (2020). Trends Chem..

[cit12] Chung W. J., Nam Y. H. (2020). ECS J. Solid State Sci. Technol..

[cit13] Zhong J., Chen D., Zhao W., Zhou Y., Yu H., Chen L., Ji Z. (2015). J. Mater. Chem. C.

[cit14] Huang J., Hu X., Shen J., Wu D., Yin C., Xiang R., Yang C., Liang X., Xiang W. (2015). CrystEngComm.

[cit15] Zeng P., Cao Z., Chen Y., Yin M. (2017). J. Rare Earths.

[cit16] Meng Q., Chen L., Zhang S., Huang L., Lei R., Zhao S., Xu S. (2019). J. Lumin..

[cit17] Durairajan A., Balaji D., Kavi Rasu K., Moorthy Babu S., Valente M. A., Thangaraju D., Hayakawa Y. (2016). J. Lumin..

[cit18] Liao J., Nie L., Wang Q., Liu S., Wen H. R., Wu J. (2016). RSC Adv..

[cit19] Upconverting Nanoparticles: From Fundamentals to Applications, ed. V. K. Rai, John Wiley & Sons, 2022

[cit20] Wang H., Jia G., Yang F., Wei Y., You Z., Wang Y., Li J., Zhu Z., Lu X., Tu C. (2006). Appl.
Phys. B.

[cit21] Meng Q., Chen L., Zhang S., Huang L., Lei R., Zhao S., Xu S. (2019). J. Lumin..

[cit22] Yuan N., Liu D. Y., Yu X. C., Sun H. X., Ming C. G., Wong W. H., Song F., Yu D., Pun E. Y., Zhang D. L. (2018). Mater. Lett..

[cit23] Kesarwani V., Rai V. K. (2022). J. Appl. Phys..

[cit24] Karlsson S., Bäck L. G., Kidkhunthod P., Lundstedt K., Wondraczek L. (2016). Opt. Mater. Express.

[cit25] Marzuki A. and Fausta D. E., IOP Conf. Ser.: Mater. Sci. Eng., IOP Publishing, 2019, vol. 578, No. 1, p. 012043

[cit26] Azam M., Rai V. K. (2017). Solid State Sci..

[cit27] Huang J., Zhang L., Xia L., Shen X., Wei W., You W. (2020). Opt. Mater..

[cit28] Cullity B. D. , Answers to problems: Elements of X-ray diffraction, Addison-Wesley Publishing Company, 1978

[cit29] Pattnaik S., Rai V. K. (2020). Mater. Res. Bull..

[cit30] Shannon R. D. (1976). Acta Crystallogr., Sect. A: Cryst. Phys., Diffr., Theor. Gen. Crystallogr..

[cit31] Ben Bacha F., Guidara K., Dammak M., Megdiche M. (2017). J. Mater. Sci. Mater. Electron..

[cit32] Rasu K. K., Balaji D., Babu S. M. (2016). J. Lumin..

[cit33] Prasad M., Rai V. K. (2022). Methods Appl. Fluoresc..

[cit34] Jia C. L., Li S., Song X. X. (2017). Appl. Phys. A.

[cit35] Zhang N., Chen D., Niu F., Wang S., Qin L., Huang Y. (2016). Sci. Rep..

[cit36] Prasad M., Rai V. K. (2020). J. Alloys Compd..

[cit37] Lei Y., Song H., Yang L., Yu L., Liu Z., Pan G., Bai X., Fan L. (2005). J. Chem. Phys..

[cit38] Pattnaik S., Rai V. K. (2022). Methods Appl. Fluoresc..

[cit39] Ran W., Sun G., Ma X., Zhang Z., Yan T. (2022). Dalton Trans..

[cit40] Pattnaik S., Mondal M., Mukhopadhyay L., Basak S., Rai V. K., Giri R., Singh V. (2022). New J. Chem..

[cit41] Haouari M., Maaoui A., Saad N., Bulou A. (2017). Sens. Actuators, A.

[cit42] Kumar Soni A., Kumar Rai V., Kumar S. (2016). Sens. Actuators, B.

[cit43] Haouari M., Maaoui A., Saad N., Bulou A. (2017). Sens. Actuators, A.

[cit44] Xu L., Liu J., Pei L., Xu Y., Xia Z. (2019). J. Mater. Chem. C.

[cit45] Prasad M., Rai V. K. (2022). Methods Appl. Fluoresc..

[cit46] Pandey A., Rai V. K., Kumar V., Kumar V., Swart H. C. (2015). Sens. Actuators, B.

[cit47] Jiang Y., Tong Y., Chen S., Zhang W., Hu F., Wei R., Guo H. (2021). Chem. Eng. J..

[cit48] Dutta S., Som S., Chen T. M. (2018). ACS.Omega.

[cit49] Gao J., Ren X., Yang K., Zhao S., Huang L., Xu S. (2021). Optik.

[cit50] Zou Z., Wu T., Lu H., Tu Y., Zhao S., Xie S., Han F., Xu S. (2018). RSC Adv..

[cit51] Meng Q., Chen L., Zhang S., Huang L., Lei R., Zhao S., Xu S. (2019). J. Lumin..

[cit52] Zhang Q., Wang X., Wang Y. (2021). J. Alloys Compd..

[cit53] Wen D., Liu H., Guo Y., Zeng Q., Wu M., Liu R. S. (2022). Angew. Chem., Int. Ed. Engl..

[cit54] Yang Y., Fu L., Ren X., Zhu Y., Zhu J., Wu Y., Pu X., Zhang Y. (2021). J. Lumin..

[cit55] Mukhopadhyay L., Rai V. K. (2020). Mater. Res. Bull..

[cit56] Liang Y., Zhang Y., Yang H., Zhang Y., Zhang J., Wang L., Liang X., Zhong J., Xiang W. (2021). Appl. Phys. Lett..

[cit57] Kim Y. H., Arunkumar P., Im W. B. (2015). Ceram. Int..

[cit58] Lee Y. K., Lee J. S., Heo J., Im W. B., Chung W. J. (2012). Opt. Lett..

[cit59] Zhang X., Yu J., Wang J., Lei B., Liu Y., Cho Y., Xie R., Zhang H., Li Y., Tian Z., Li Y., Su Q. (2017). ACS Photonics.

[cit60] Janulevicius M., Marmokas P., Misevicius M., Grigorjevaite J., Mikoliunaite L., Sakirzanovas S., Katelnikovas A. (2016). Sci. Rep..

[cit61] Kesarwani V., Rai V. K. (2022). Opt. Laser Technol..

[cit62] Chen J., Zhang N., Guo C., Pan F., Zhou X., Suo H., Zhao X., Goldys E. M. (2016). ACS Appl. Mater. Interfaces.

